# Infectious Diseases Hospital, Alloa

**Published:** 1895-06-08

**Authors:** 


### EDITOR'S LETTER-BOX.

[Our correspondents are reminded that proxility is a great bar to publication, and that brevity of style and conciseness of statement greatly facilitate early insertion.]

### INFECTIOUS DISEASES HOSPITAL, ALLOA.

The architects, Messrs. Melvin and Son, write from Mar Street, Alloa: We observe that in your issue of June 1st you have a short notice of the new Infectious Diseases Hospital, at Alloa, and therein you state that the disinfector is by Manlove, Alliot, and Co., Nottingham, whereas it should have been stated to be the Washington Lyon make.

